# Mentorship in academic radiology: why it matters

**DOI:** 10.1186/s13244-019-0799-2

**Published:** 2019-11-15

**Authors:** Miriam A. Bredella, David Fessell, James H. Thrall

**Affiliations:** 1 Department of Radiology, Massachusetts General Hospital and Harvard Medical School, Yawkey 6E, 55 Fruit Street, Boston, MA 02114 USA; 2 Musculoskeletal Division, Taubman Center, University of Michigan, Room 2910K, 1500 E Medical Center Drive, SPC 5326, Ann Arbor, MI 48109 USA

**Keywords:** Mentoring, Radiology, Faculty, Burnout, Job satisfaction

## Abstract

Mentorship plays a critical role in the success of academic radiologists. Faculty members with mentors have better career opportunities, publish more papers, receive more research grants, and have greater overall career satisfaction. However, with the increasing focus on clinical productivity, pressure on turn-around times, and the difficult funding climate, effective mentoring in academic radiology can be challenging. The high prevalence of “burnout” among radiologists makes mentorship even more important. This article reviews benefits and challenges of mentorship in academic radiology, discusses how to institute a faculty mentoring program, examines different types of mentoring, and reviews challenges related to diversity and inclusion.

## Key points

- Mentorship plays a critical role in the success of academic radiologists
- Mounting pressures on academic radiologists in a changing healthcare environment can lead to physician burnout
- Mentoring is an effective means to achieve career satisfaction and help prevent burnout
- Establishing a mentoring program in academic radiology requires the identification and training of effective mentors, and mentees benefit from multiple mentors for different needs
- Women and underrepresented minorities in medicine often do not have access to mentors but benefit most from a mentoring relationship

## Introduction

Mentorship plays a critical role in the success of academic radiologists. A mentor helps a mentee reach both personal and career goals by functioning as an adviser and role model while providing support and sharing knowledge [1]. Multiple studies have shown that faculty members with mentors have better career opportunities, publish more papers, are more likely to receive research grants, and have greater overall career satisfaction [2–6]. The benefits of mentorship go both ways: while mentees benefit from professional support, advice, and career-specific skills, mentors receive intellectual and professional stimulation, personal enrichment, and a sense of giving back to their institutions and specialty [7, 8]. Moreover, reverse mentoring, where mentees provide skills and guidance to their mentors, can be beneficial to the mentor, for example in the use of social media, often more familiar to younger mentees [9]. At an institutional level, mentorship offers the opportunity to develop future leaders in clinical care, research, education, and administration [8], and institutions with successful mentoring programs have better clinical and academic productivity, better retention and academic promotion rates [10, 11].

While most radiology residency programs have incorporated formal mentoring programs for residents [4, 5, 12–14], fewer departments have instituted mentoring programs for junior faculty [15, 16]. Junior or newly hired faculty in academic radiology face specific obstacles including new clinical, educational, and research responsibilities, often paired with personal changes and growing family demands [16, 17]. In addition, the increasing focus on clinical productivity, pressure on turn-around times, and demands on non-interpretive tasks, such as consultations, administration, meetings, and teaching [18–20], have led to a high prevalence of “burnout” among radiologists [21], making mentoring of junior faculty members even more important.

This article reviews the benefits and challenges of mentorship in academic radiology, discusses how to institute a faculty mentoring program, examines different types of mentoring, and reviews challenges related to diversity and inclusion.

## Why mentorship in academic radiology matters more than ever

A career in academic radiology requires balancing competing demands from clinical work, education, and research. Reasons for junior faculty to embark on a career in academic radiology include opportunities for teaching, research, and having an interesting mix of clinical cases, while deterrents include insufficient financial compensation, lack of academic time, high clinical workload, and the long time for promotion [22]. Most faculty who leave academics do so within the first years; therefore, efforts should be concentrated on retaining junior faculty through effective mentoring to address the following challenges.

### Mounting pressures on academic radiologists in a changing healthcare environment

Academic radiology has experienced major changes over the past decades [23], with increasing imaging volumes coupled with lower reimbursement rates [24], pressure on shorter turn-around times (TAT), and growing demands on non-interpretative tasks [18, 19, 25]. The average workload of radiologists as measured by annual relative value units (RVUs) per radiologist has increased by 70% over the last 25 years [18], and this increase was coupled with lower TAT. In fact, low TAT has been among one of the highest priorities in academic medical centers [20]. In addition to the increasing interpretative demands, non-interpretative demands, such as consultations, presentations during multidisciplinary conferences, meetings, training of technologists, and requirements for resident supervision, are rising. A study by Dhanoa et al. has shown that radiologists spend only about half of their time on clinical activities and the remaining time is spent on non-interpretive tasks [19]. Furthermore, decreasing autonomy associated with practice standardization, structured reporting, evidence-based recommendations, procedural checklists, and increasing dependence on integrated information technology systems [26], place academic radiologists at an increased risk of burnout [21, 27]. These challenges indicate the need for dedicated mentoring of radiology faculty as mentoring has been shown to help prevent burnout in radiology [28, 29].

### Challenges of junior faculty members to focus on academic career development

Junior and new faculty members typically focus on managing the clinical workload, trying to “survive” in a new hospital system with new colleagues and often growing family responsibilities [17], with little emphasis on their academic career development. Because of economic pressures, junior faculty members usually have less protected academic time compared to their senior peers [30]. In these circumstances, mentorship becomes critical in helping junior faculty to improve their clinical efficiency and to guide them in their professional and personal development, including academic promotion [31]. Academic promotion is an important goal for junior faculty. Promotion not only represents a formal marker of academic success but may also lead to additional salary and benefits [30]. Junior faculty members often lack the knowledge of promotion criteria within their institution and benefit from mentoring to better identify their academic track and area of expertise, guidance on which academic activities to focus, and advice on whether they are ready to be promoted. In fact, 98% of academic physicians cited a lack of mentoring as the largest or second largest factor in hindering career progress [32].

### Grant funding climate

While most academic radiologists who do research are “clinician scholars” who are not dependent on external funding, a small percentage of academic radiologists are able to obtain government or industry-funded grants [30]. Such funding supports the development, testing, and dissemination of innovative new imaging technologies and is crucial to advance our field [33, 34]. The primary source of grant funding in the USA is the National Institutes of Health (NIH). NIH grants are highly competitive, and only a small number of NIH grant applications receive funding [35]. Junior faculty without prior track records of research success face a steep challenge in getting past the threshold of obtaining their first grants, an important step in establishing independent research careers. Declining funding combined with a shift to multidisciplinary, collaborative team-science, requires mentoring programs to support early-stage investigators [36–38]. Mentors with track records of success in obtaining grants are invaluable in helping junior faculty in understanding the ins and outs of grant structure and grant writing and grantsmanship.

### Lack of training in professionalism

Over the last decade, there has been an increasing focus on professionalism in academic medicine. Teaching and assessing competence in professionalism is now an explicit expectation for Accreditation Council Graduate Medical Education (ACGME) accredited programs in radiology [39]; however, this topic often receives little attention beyond residency. Unprofessional conduct by radiologists, including conduct leading to prison sentences, has emphasized the need for education and mentorship in professional conduct. Over the past years, radiologists have pled guilty to bribery, fraudulent billing, unlawful prescription of controlled substances, and submission of fraudulent expense reports [40]. This highlights the need for discussions around professionalism by mentors and role models beyond compliance with policies and procedures [40].

Generational differences, increased diversity in the workforce, and the use of social media can also add to misunderstandings of professional conduct [41]. Moreover, the patient-doctor relationship has changed from paternalistic medicine to patient-centered, with patients being encouraged and empowered to control the direction of their care [41, 42]. The use of the Internet, websites, chat rooms, and blogs by patients and their families, to acquire and share information, can pose challenges in professionalism. Radiologists need to behave appropriately and respectfully in digital and social media, and mentors are needed more than ever to teach the appropriate use of social media, patient confidentiality, and digital professionalism [41].

The current healthcare climate poses several challenges to academic radiologists. Successful mentoring is more important than ever for retaining and managing the current and upcoming demands of junior faculty, and to ensure the future of radiology [7].

## Establishing a faculty mentoring program in academic radiology

Several studies have examined key components of a successful mentoring program in academic radiology [3, 7, 8, 15, 43]. A mentoring program must be customized to meet the specific needs of the faculty to make sure that the experience is rewarding for both mentors and mentees, rather than an obligation [15]. The identification and training of potential mentors are crucial for the success of a mentoring program [44, 45]. An important concept of mentoring in academic radiology is that one mentor cannot do it all and mentees need to assemble a mentorship team [46]. In our mentoring program for junior faculty in the department of radiology at the Massachusetts General Hospital (MGH), we encourage mentees to create a mentorship “Board of Directors,” tailored to the unique needs of each faculty member. A diverse group of mentors can provide the mentee with a wide range of perspectives related to research, administration, and clinical expertise and allows networking and development of mentorship relations when one does not need anything in return. Mentees are also encouraged to reach out to mentors from different departments and institutions, also outside of medicine. For this purpose, the Department of Radiology at MGH provides funds for mentees to take potential mentors out to lunch or dinner. This support has eliminated a financial barrier to less formal, more social mentor-mentee interactions, strengthening relationships.

Setting clear expectations for mentors and mentees, reviewing a mentorship agreement which states the ground rules of the mentorship relationship, and creating a career development plan with short- and long-term goals, which can be revisited during the mentoring meetings, are key components of our mentoring program. Additional goals of the program are academic promotion and preparing mentees for the annual career conference with their division heads. In this regard, mentors and mentees receive dedicated lectures and written material on promotion criteria and how to make the best of their annual career conference.

Regular meetings among the mentees of our program help to exchange ideas and allows for peer-mentoring. We also provide funds to help junior faculty members to hire medical students or college students to help with research and scholarly projects. These interactions give the mentees the opportunity to become mentors themselves for college or medical students.

Finally, assessment of the overall effectiveness of a mentoring program can be obtained through evaluations on career satisfaction, attrition rates of junior faculty, promotion, research-related metrics, such as the number of scientific publications, scientific talks, and grant submissions, teaching-related metrics, such as the number of courses taught and educational publications, and clinical-related metrics, including the development of new clinical care and participation in clinical committees. Feedback from these evaluations can help radiology departments to understand the value of mentorship and allows them to tailor their resources to provide effective support to junior faculty.

### Mentors

The identification and preparation of effective mentors are key to the establishment of a successful mentoring program [3, 43]. Several studies have explored the characteristics of good mentors and interactions with their mentees [3, 32, 47]. Good mentors should be honest, collaborative, and accessible. A well-established position within the academic community that allows professional and networking opportunities for the mentee, professional, and life experiences that can guide mentees in academic and personal decisions, are additional key attributes of a good mentor. Mentors are also expected to provide guidance in dealing with colleagues and institutional “politics” [31]. Mentors should actively seek feedback from the mentee to determine what is working in the relationship and what needs to be addressed [4]. Preparation and training of potential mentors include the distribution of written material and participation in mentorship-focused seminars [11].

The main barrier to becoming a mentor is lack of time [11, 14, 16], often restricting the number of mentees a mentor can have to 2 or 3 [13]. A potential downside for mentors is the time spent on mentoring projects which may be less relevant to the mentor, taking time away from performing more advanced projects, necessary to advance the mentor’s career [48]. Including alumni as mentors who do not face the pressures of internal faculty, can help to increase the pool of mentors [14]. Providing protected time and resources to mentors will increase the likelihood of success and sustainability of a mentoring program [11]. Formal recognition of mentors for their time and effort and an emphasis on mentoring during the annual career conference and assessment for academic promotion will also help to motivate potential mentors to participate in mentoring [16].

Malignant mentor behaviors have been described by Chopra et al., which include taking the mentees’ ideas and labeling them as their own, exploiting the mentee by giving them low-yield activities that are not advancing their careers, or dominating the mentee, not allowing input from others, which can lead to isolation of the mentee [49]. Those behaviors are often due to distraction, time constraints, and avoiding feedback, even if constructive criticism is necessary [49]. Many of these problems can be resolved or avoided through mentor and mentee education and open communication and the creation of a mentorship team. However, if necessary, mentees should not be afraid and be supported to change mentors if their needs are not met [8, 32].

### Mentees

Mentees play a critical role in the success of the mentor-mentee relationship, and mentees need to be instructed about what is expected from them. This can be accomplished through seminars and the distribution of written material prior to entering into a formal mentoring relationship. Studies have indicated that mentees should be proactive and should take initiative for the mentoring relationship [3, 50]. They should be prepared with an outline of the topics they would like to discuss, complete tasks that were agreed upon, and be receptive to feedback [3, 51]. The goal for the mentee is to advance personally and professionally, remain independent and goal-oriented.

Mentee missteps often stem from uncertainty about their responsibilities and the mentors’ failure to address them. Vaughn et al. described common mentee missteps and solutions on how to overcome them [52]. Awareness of these pitfalls and proactive mentee education can not only prevent failure but also boost the progress of the mentor-mentee relationship [52].

### Matching mentors and mentees

Identifying helpful mentors for junior and newly hired faculty is key to a successful transition to a new faculty position [8]. However, identifying mentors can be challenging for young and new faculty members who may have limited interactions with senior colleagues beyond their own division [16]. For these reasons, early implementation of mentorship strategies and identification of mentors will help improve the integration of junior and newly hired faculty [8, 16, 43, 53].

Several ways to match mentors and mentees have been described, ranging from self-selected to assigned mentors [54]. The importance of having the right “chemistry” has been stressed by participants in mentoring relationships [32] and participants were more willing to engage in a mentoring program if the personalities matched [55]. Some programs pair mentees with mentors within their own department and division, whereas others, especially smaller programs, suggest mentorship outside the department and division, and even outside of the institution [11].

In our radiology mentoring program at the MGH, we pair junior faculty members with mentors outside of their division. Mentees often already have mentors within their division and having mentors in the same division can lead to conflicts of interest regarding shared or needed resources or when conflicts arise within the division. Pairing mentors and mentees across divisions also fosters better communication within the department. Pairing of mentors and mentees occurs with input from the mentor and mentee with guidance from the director of the mentoring program. The director of the mentoring program meets with each mentee and suggests potential mentors based on the right chemistry and needs. Both, mentors and mentees, have input in the final pairing.

Some programs prefer a more natural matching process by which mentees independently choose their own mentors [11, 15]. Mentees can be paired with a single mentor [15] or choose several mentors [56]. While we pair the mentee with a single mentor primarily based on chemistry, mentees are encouraged to reach out to other mentors and create a mentorship “Board of Directors,” tailored to the unique needs of each faculty member. Developing a network of mentors within and outside of the department, and even outside of medicine, such as in business or government, may help junior faculty members to gain diverse perspectives related to radiology, health care, and administration. Active membership in national radiology societies can also provide junior faculty members with a network for connecting with mentors worldwide [16].

### Peer mentoring

While mentorship is often viewed as a dyad with a senior mentor and a junior mentee, peer-mentoring, which encourages junior faculty members of similar rank to collaborate and exchange ideas, is an important part of a mentoring program. Because of the inherent equality of the participants who are often at similar stages in their personal life, participants are often more comfortable discussing professional and non-professional topics, such as the balance between work and family [57]. Moreover, these relationships offer more personal feedback and friendship than traditional mentoring relationships [8]. Peer-mentoring has been shown to improve support, collaboration, and access to resources [58, 59]. Therefore, mentoring programs should encourage radiologists to identify peers within and outside of the department and create formal opportunities for peer-mentoring.

### Speed mentoring

Speed mentoring is an organized event in which a junior faculty member is paired with six senior faculty members for a series of 10-min encounters [60]. A speed mentoring event was offered during the Association of University Radiologists (AUR) Annual Meeting, to improve access to mentorship for clinician educators in radiology [61]. The speed mentoring event created the opportunity for mentors and mentees from geographically diverse academic institutions to network with each other. In addition, mentees had the opportunity to reach out for additional remote mentorship beyond the speed mentoring event. Following the event, both the mentors and mentees felt a stronger sense of community and inclusiveness and felt more connected to the AUR [61].

## Mentoring underrepresented minorities in medicine and women

Women are underrepresented in the radiology workforce and women only occupy a minority of leadership positions without a significant change over the last decade [62, 63]. The paucity of female leaders in radiology results in a lack of role models and deters women to choose radiology as a career [64].

Female faculty members are less likely to advance academically than their male colleagues of comparable seniority [65]. Demands in clinical workload combined with family obligations and lack of mentoring are factors that hamper the academic careers of women [66, 67]. A major barrier to the academic advancement of women is a lack of role models and mentors, however, mentorship is particularly crucial for women to achieve academic promotion and advance their professional careers [68]. However, the number of women interested in mentorship often outnumbers the number of women who are available or feel qualified [69]. Although women, in general, do not seem to prefer female mentors, they may seek out female mentors for specific advice, including on how to best juggle work and family responsibilities, especially in fields where women are still in the minority, such as radiology [70]. Innovative traditional and peer-mentoring programs to promote gender equity have been developed [57, 63, 68, 69], such as the Leadership Intervention to Further the Training of Female Faculty (LIFT-OFF) aimed at women radiologists to achieve academic success and assume leadership positions [63].

Mentoring is particularly important for the success and retention of faculty underrepresented in medicine (URiM) [71, 72] who may have limited access to mentors and often face additional challenges, such as bias, prejudice, lack of confidence, and the feeling of isolation, which can lead to attrition from academic careers [73]. For URiM faculty, finding a mentor of similar background is challenging. The race is often cited as an obstacle for mentoring, and URiM faculty reported difficulties in explaining their own racial context to a non-minority mentor [74].

Radiology departments should identify a diverse group of mentors who are able to meet the needs of a diverse faculty. Providing a network of diverse mentors is key to a successful and inclusive mentoring program that is able to provide equal opportunities and advancement to all faculty.

## Conclusion

Mentorship in academic radiology is more important than ever given the mounting pressures of a changing healthcare environment. Mentorship for junior faculty promotes job satisfaction and career advancement, helps avoid burnout, and increases faculty retention. While mentorship is traditionally viewed as a senior mentor and a junior mentee, diverse mentorship teams and peer-mentoring are important components of a successful mentoring program. Academic institutions should recognize the value of mentoring and ensure equal opportunities and advancement of a diverse faculty.

## Data Availability

Not applicable

## Abbreviations

ACGME: Accreditation Council Graduate Medical Education
AUR: Association of University Radiologists
LIFT–OFF: Leadership Intervention to Further the Training of Female Faculty
MGH: Massachusetts General Hospital
RVUs: Relative value units
TAT: Turn-around time
